# Stachydrine targeting tumor-associated macrophages inhibit colorectal cancer liver metastasis by regulating the JAK2/STAT3 pathway

**DOI:** 10.3389/fphar.2025.1514158

**Published:** 2025-02-05

**Authors:** Yang Gui, Gengchen Xue, Yuyi Yuan, Jingbo Wang, Shuangjiao Deng, Fei Gao, Yushi Tian, Zhiqiang Zhao, Heng Fan

**Affiliations:** Department of Integrated Traditional Chinese and Western Medicine, Union Hospital, Tongji Medical College, Huazhong University of Science and Technology, Wuhan, China

**Keywords:** colorectal cancer liver metastasis, stachydrine, tumor-associated macrophages, JAK2/STAT3 signaling pathway, immunotherapeutic

## Abstract

**Introduction:**

Colorectal cancer (CRC) represents the third most prevalent form of cancer worldwide, with liver metastasis representing a significant contributor to mortality. The interaction between tumor-associated macrophages (TAMs) and tumor cells plays a pivotal role in the development of colorectal cancer liver metastases (CRLM) and represents a promising avenue for therapeutic intervention. Stachydrine (STA), a compound derived from the Leonurus heterophyllus plant, has been shown to effectively inhibit tumor growth through a range of mechanisms.

**Methods:**

The study employed imaging and histopathology to evaluate the efficacy of STA monotherapy in preventing CRLM. The inhibition of M2 macrophage polarization by STA was confirmed through the use of flow cytometry and immunofluorescence. Subsequently, a series of assays, including quantitative reverse transcription polymerase chain reaction (qRT-PCR), flow cytometry, scratch, invasion, and tube formation assays, were conducted to confirm STA’s capacity to impede tumor cell migration, invasion, and angiogenesis *in vitro*. Western blotting and flow cytometry were employed to elucidate the mechanisms through which STA exerts its effects on tumor metastasis.

**Results:**

In our research, STA has been shown to attenuate liver metastasis in CRC mouse models by inhibiting the polarization of macrophages to the M2 phenotype. This anti-metastatic effect is dependent on the presence of macrophages. *In vitro*, STA has been found to impede tumor cell migration, invasion, and angiogenesis by preventing TAMs from polarizing to the M2 phenotype via the JAK2/STAT3 signaling pathway. Moreover, the combination of STA with anti-PD-1 therapy has been observed to restore immune infiltration within the tumor microenvironment and inhibit tumor progression.

**Conclusion:**

The findings of this study demonstrate that STA exerts an inhibitory effect on colorectal cancer liver metastasis by targeting macrophages and impeding their M2 polarization via the JAK2/STAT3 pathway. Furthermore, the combination of STA with anti-PD-1 therapy has been observed to enhance the effectiveness of immune checkpoint blockade and reduce tumor spread, indicating the potential of STA to improve the efficacy of immunotherapy for liver metastases.

## Graphical Abstract

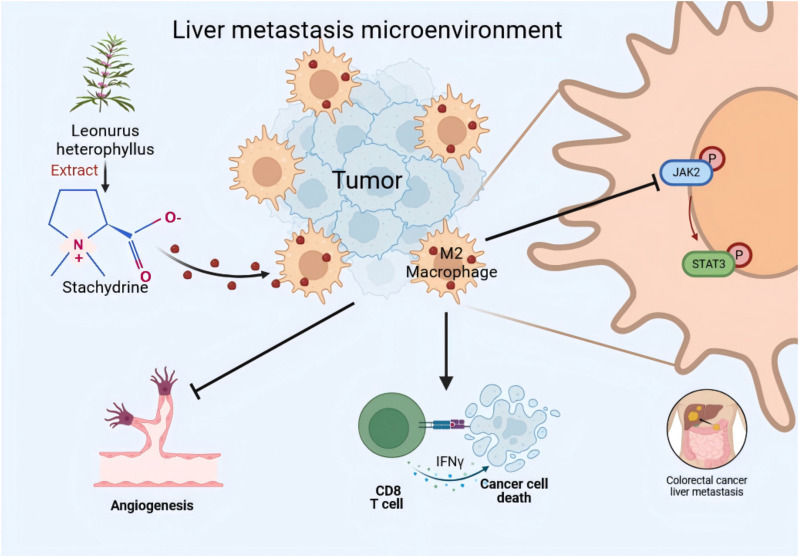

## 1 Introduction

Colorectal cancer (CRC) had 1,926,118 new cases in 2022, accounting for 9.6% of all new cancer cases globally and ranking third in terms of incidence. In terms of mortality, 903,859 deaths were attributed to CRC in 2022, with a proportion of 9.3%, ranking second (Bray et al., 2024). The primary factors contributing to patient mortality are distant metastasis and recurrence. Indeed, over half of CRC-related deaths are attributed to metastatic dissemination to the liver (Xiang et al., 2022). Patients with colorectal cancer liver metastasis (CRLM) frequently present with an immunosuppressed microenvironment and experience limited efficacy from immunotherapy (Li et al., 2023). Consequently, it is of the utmost importance to investigate novel therapeutic strategies to reverse the immunosuppressive tumor microenvironment (TME) for the treatment of CRLM.

The process of cancer metastasis is influenced by the intricate alterations that occur within cancer cells and the complex interactions between cancer cells and their surrounding microenvironment. The TME represents a highly organized and diverse ecosystem, encompassing tumor cells alongside non-cancerous components such as endothelial cells, fibroblasts, stromal cells, adaptive immune cells, and innate immune cells. Furthermore, the TME encompasses a dynamic extracellular matrix, which interacts and influences the tumor microenvironment in a complex manner (Lv et al., 2021; Hou et al., 2024). Macrophages play a significant role in the TME and are intricately involved in the development and metastasis of CRC. These cells exhibit remarkable adaptability to diverse environments and treatments, giving rise to two distinct subtypes: M1 (classically activated) and M2 (alternatively activated) macrophages are induced in response to external stimuli (Wang et al., 2024; Zhou et al., 2024) It is noteworthy that both subtypes are prevalent within the TME. However, in CRC, TAMs frequently polarize towards the M2 phenotype, which is associated with immunosuppression and tumor progression (Xu et al., 2023). M2-TAMs secrete a series of cytokines, including platelet-derived growth factor, epidermal growth factor, vascular endothelial growth factor A, platelet-derived growth factor, transforming growth factor-β, interleukin-8, and others. These factors have been demonstrated to stimulate tumor production and proliferation, promote angiogenesis in the tumor area, provide an abundant blood supply and nutrition to the tumor, and facilitate tumor metastasis (Hughes et al., 2015; Williams et al., 2016). In addition, M2-TAMs secrete a considerable number of matrix metalloproteinases and cathepsins, which facilitate the degradation of the extracellular matrix and basement membrane (Fu et al., 2020). These processes contribute to tumor metastasis. Consequently, the targeting of M2-TAMs represents a promising and efficacious strategy for the management of CRLM.

Recent research indicates that immunosuppression plays a pivotal role in the pathogenesis of CRLM and immune tolerance (Zhang et al., 2020). Prior studies have demonstrated that (Programmed Death-Ligand 1) PD-L1 expression is elevated in liver metastases compared to primary CRC, indicating the presence of a distinct microenvironment that may facilitate immune evasion (Wei et al., 2020). Immune checkpoint blockade (ICB) therapy has demonstrated effectiveness in managing advanced malignancies, including melanoma, non-small-cell lung cancer, and renal cell carcinoma. However, its efficacy in treating colorectal cancer liver metastases remains constrained (Thomas et al., 2023). M2-phenotype TAMs are composed of arginase 1 (Arg1), which hydrolyses arginine. The depletion of arginine has been demonstrated to affect the activation and proliferation of T cells, leading to immunosuppression. In addition, M2-phenotype TAMs can impede the anti-tumor effect of T cells and facilitate the formation of an immunosuppressive TME by recruiting FOXP3^+^ regulatory T cells in a hypoxic microenvironment (Qiu et al., 2023). In conclusion, M2-TAMs can facilitate tumor growth and metastasis by releasing various pro-tumorigenic factors, and also contribute to the formation of an immunosuppressive microenvironment conducive to tumor development by regulating the function of other immune cells. Consequently, M2-TAMs are closely associated with a range of malignant events in cancer. Reversing the immunosuppressive tumor microenvironment by targeting TAMs and enhancing the efficacy of immunotherapy for colorectal cancer liver metastases is expected to become the next frontier of tumor immunotherapy.

Natural compounds extracted from plants exhibit unique pharmacological or biological activities and represent a potential source for novel therapeutics targeting various diseases, including tumors. The emergence of certain anticancer drugs like paclitaxel and vincristine from plants has provided an endless resource for the discovery and development of anticancer agents (Wang et al., 2023; Zhao et al., 2022). Stachydrine (STA), a prominent active compound found in Leonurus heterophyllus, has been extensively studied for its anticancer properties (Liu et al., 2024). A substantial body of research has demonstrated the substantial inhibitory effects of STA on a wide range of cancers, including astrocytoma (Liu et al., 2018), prostate cancer (Rathee et al., 2012), breast cancer (Zhai et al., 2024), esophageal squamous cell carcinoma (Isozaki et al., 2014), chronic myeloid leukemia (Gu et al., 2022), and hepatocellular carcinoma (Bao et al., 2022). The anticancer activity of STA is primarily associated with the inhibition of cell proliferation, induction of apoptosis, and blockade of cell migration and invasion. These effects are achieved through modulation of multiple molecular pathways, thereby providing a robust mechanistic foundation for their potential therapeutic applications in a range of cancers. Nevertheless, the molecular mechanisms that underpin the efficacy of STA in the treatment of CRLM remain unclear. Furthermore, the potential inhibitory effects of STA on tumor progression and metastasis through modulation of the immunosuppressive tumor microenvironment remain uncertain. Consequently, it is essential to investigate the impact and potential molecular pathways of STA on CRLM both *in vitro* and *in vivo*, as well as to devise novel therapeutic approaches for this condition.

## 2 Materials and methods

### 2.1 Colorectal cancer liver metastasis model

Male 6 - week - old C57BL/6J mice were purchased from Wuhan Hualianke Biological Co., Ltd. After acclimatization for 1 week, mice were divided into 4 groups (n = 6/group). The mice were anesthetized with 1% pentobarbital sodium and intrasplenically injected with Luciferase - MC38 cells (2 × 10^6^) to establish the liver metastasis model. The control group received saline, and the STA - L, STA - M, and STA - H groups were orally administered Stachydrine at doses of 15 mg/kg/d, 30 mg/kg/d, and 60 mg/kg/d respectively, based on literature (Bao et al., 2022). After modeling, the mice were orally administered every other day for 3 weeks. At the end of the study, the liver weight was recorded, and liver tissues was collected for further analysis. For macrophage depletion, anti-CSF1R antibody was intraperitoneally injected on days 6, 9, 12, 15, and 18 post-tumor cell inoculation, with an initial dose of 400 μg/mouse and subsequent maintenance doses of 100 μg/mouse. In the anti-PD-1 combination therapy, 100 μg of anti-PD-1 antibody was intraperitoneally injected on days 7, 9, 11, 13, 15, 17, and 19 post-tumor cell inoculation.

### 2.2 Cell culture

Luc - MC38 cells from ATCC, HUVECs from ATCC, and THP - 1 cells from Procell were cultured in DMEM containing 10% FBS and 1% penicillin - streptomycin at 37°C in a 5% CO₂ humidified incubator. The medium with fresh FBS was replaced every other day.

### 2.3 Small animal *in vivo* imaging

After intraperitoneal injection of 1% sodium pentobarbital anesthesia in mice, D-luciferin potassium salt was intraperitoneally injected. After a 10-minute waiting period, spontaneous signals within the mice were detected using a small animal live imaging system (Bruker, Germany).

### 2.4 Tissue flow cytometry

Tumor tissue (100 mg) was digested at 37°C for 1 h in a solution of collagenase V, hyaluronidase, and DNase I, agitated every 15 min. After filtration (70 µm), it was incubated in RBC lysis buffer on ice for 5 min and washed twice with PBS. For T cell cytokine detection, single cells were cultured 6 h in RPMI 1640 + 10% FBS, stimulated with Leukocyte Activation Cocktail. Before staining, 1 × 10^6^ cells were blocked with mouse Fc receptor blocker for 20 min, then surface stained. After fixation and permeabilization, intracellular staining was done with the Kit. Data was analyzed with CytExpert software.

### 2.5 Immunofluorescence

Fresh tumor tissue was fixed in 4% PFA, paraffin-embedded, and sliced into 4-μm sections. After deparaffinization, hydration, and antigen retrieval, slides were blocked with 5% BSA. Primary Abs were incubated at 4°C overnight, then secondary Abs for 1 h at RT. Nuclei were stained with DAPI for 10 min. Slides were scanned with a panoramic scanner (Pannoramic MIDI, 3DHISTECH).

### 2.6 Bone marrow-derived macrophages culture and macrophage polarization

Bone marrow cells were isolated from the femurs and tibias of male C57BL/6J wild-type mice aged 6–7 weeks. Following the protocol described previously (Zhou et al., 2024), the cells were differentiated into mature macrophages using macrophage colony-stimulating factor (M-CSF). Subsequently, they were induced into M2 macrophages using IL-4 and stimulated for 24 h.

### 2.7 Preparation of conditioned medium

Colon cancer cells were cultured in Dulbecco’s modified eagle medium (DMEM) to 80% confluency, washed twice with PBS, and incubated in 10 mL DMEM +1% FBS for 48 h. The medium was collected, centrifuged, and filtered. The tumor-primed medium was prepared by mixing the conditioned medium with the regular medium at 1:1 (v/v).

### 2.8 Reverse transcription quantitative polymerase chain reaction

RNA was extracted from cells with RNAiso Plus. cDNA was synthesized using the HiScript III 1st Strand cDNA Synthesis Kit. Gene expression changes were analyzed by real-time fluorescence quantitative PCR (Applied Biosystems) with the RT-PCR kit. Relative mRNA levels were calculated by the comparative CT method, with the control group average set as 1. Results were expressed as relative mRNA expression levels. Primer sequences are in Supplementary File S1.

### 2.9 Wound healing assay

DMEM with or without STA was added to the co-culture and incubated for 24 h. When cell confluency in the lower chamber was ∼70%, scratches were made with a pipette tip. The medium was replaced with serum-free DMEM. After 24 h, images were taken with an inverted microscope (IX73, OLYMPUS) and cell migration was assessed via randomly selected fields.

### 2.10 Transwell assay

CRC cells and HUVEC cells were seeded separately into the upper chamber containing Matrigel while M2 macrophages were seeded into the lower chamber of the culture plate. DMEM medium containing or lacking STA was added to the co-culture system, and the cells were incubated at 37°C for 24 h. After incubation, the invading cells were fixed, stained, photographed, and quantified.

### 2.11 Tube formation assay

M2 macrophages were seeded into the upper chamber, while HUVEC cells were seeded into the lower chamber containing Matrigel. DMEM medium containing or lacking STA was added to the co-culture system. After incubating the system at 37°C for 6 h, tube formation was observed, and photographs were taken.

### 2.12 Western blot

Cells were lysed in RIPA buffer for protein collection. Proteins were separated by SDS-PAGE and transferred to PVDF membranes. After blocking, membranes were incubated with specific antibodies. Post TBST washing, membranes were incubated with secondary antibodies. Finally, membranes were exposed to ECL reagent and imaged on an iBright 750 system.

### 2.13 Statistical analysis

The GraphPad Prism software (version 8.0) was utilized for all statistical analyses and the generation of graphical representations. For the assessment of the statistical significance of differences between two independent groups, a two-tailed unpaired Student’s t-test was applied. This test operates on the principle of comparing the means of two groups under the presumption that the variances are equal (or unequal, contingent upon the outcomes of the preliminary variance tests, which were carried out following standard statistical procedures). When it came to comparing three or more independent groups, a one-way analysis of variance (ANOVA) was utilized. In this ANOVA process, we adhered to the standard protocol, which involved computing the sum of squares between groups and within groups. For the survival analysis, the data were plotted and contrasted using the Log-Rank (Mantel-Cox) test, which is prevalently employed in survival analysis to compare the survival distributions among different groups. Unless otherwise denoted in the accompanying legend, all data were presented as mean ± standard deviation (SD). The p-values were indicated as follows: *P < 0.05; **P < 0.01; ***P < 0.001. The symbol “ns” represents “not significant.”

## 3 Results

### 3.1 STA suppresses hepatic metastasis of CRC

**FIGURE 1:**
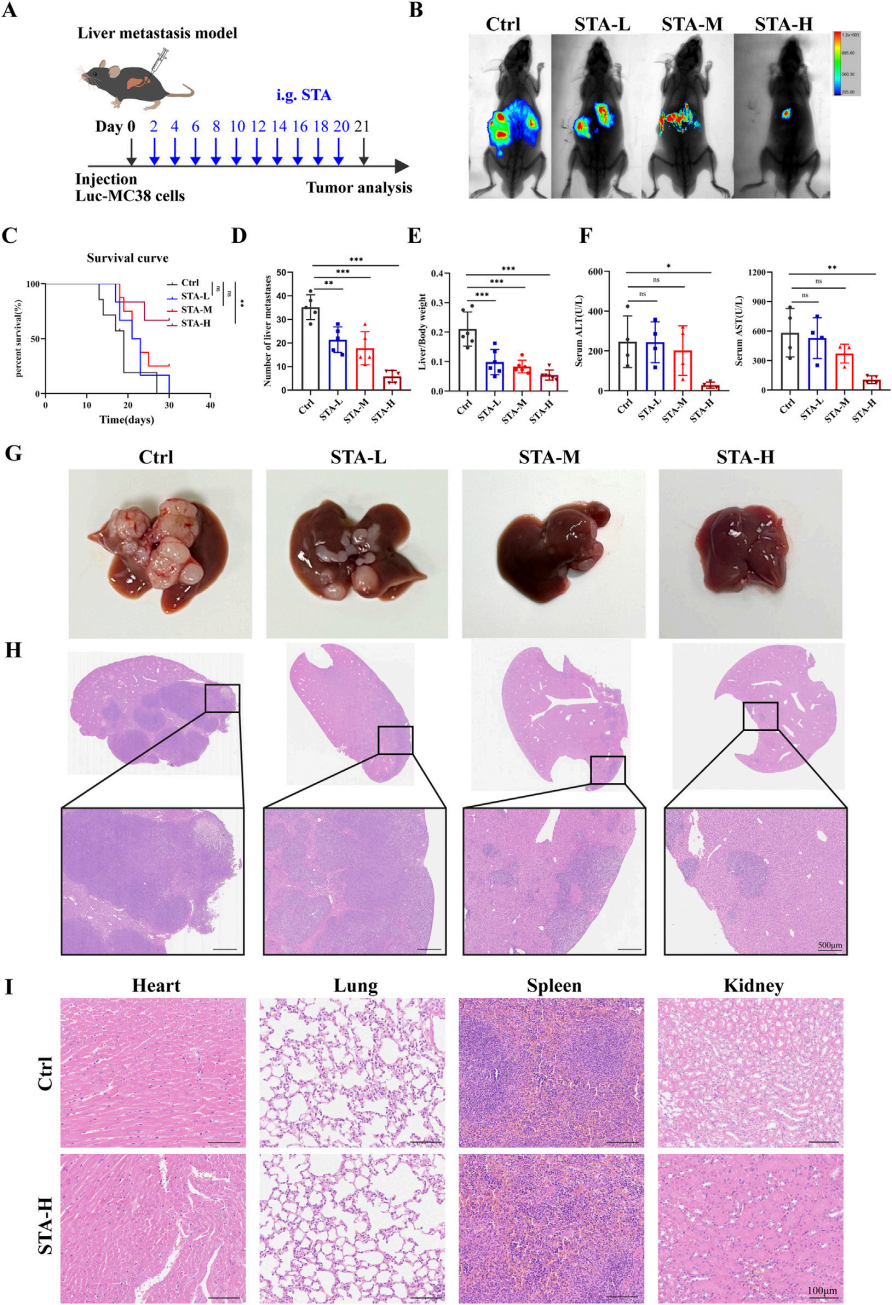
STA inhibits CRC hepatic metastasis. **(A)** The MC38-Luc cells were injected to establish a hepatic metastasis model, followed by continuous treatment with STA (i.g.) or physiological saline (i.g.) for 3 weeks; i. g., intragastrically. **(B)** Live imaging was conducted for 21 days to monitor tumor growth within the mice of each group. **(C)** Kaplan–Meier survival curves were plotted for mice in the Ctrl, STA-L, STA-M, and STA-H groups (n = 6); the Log-Rank (Mantel-Cox) test was utilized to assess the data (**p < 0.01 vs. the Ctrl group). **(D)** Number of liver metastases. **(E)** Liver/body weight. **(F)** Peripheral blood ALT and AST level. **(G)** Representative images of the liver surface of mice from each group were captured. **(H)** Hepatic metastatic foci formation was observed using HE staining, with a scale bar of 500 μm. **(I)** Representative images of HE staining of the heart, lungs, spleen, and kidneys of mice treated with STA-H (60 mg/kg/day) and saline were obtained. Scale bar: 100 μm. Data are presented as mean ± standard deviation (mean ± SD), n = 3–4.

The liver represents the primary site of metastasis for CRC. To examine the impact of STA on CRC metastasis *in vivo*, a mouse model of hepatic metastasis was developed. Firstly, C57BL/6 mice were injected with Luc-MC38 cells into the spleen. After 21 days of treatment, hepatic metastatic tumors were detected by live animal imaging (Figure 1A). The results of live animal imaging demonstrated a notable reduction in both the number and size of hepatic metastatic foci following STA treatment (Figure 1B). Furthermore, the overall survival of mice in the STA treatment group was prolonged in comparison to the control group. Notably, the survival of mice in the low and intermediate dose groups was not statistically different from that of the control group, whereas only the survival of mice in the high dose group was statistically different from that of the control group (Figure 1C). In the liver metastasis model, the number of liver metastases in the STA group was significantly lower than in the control group, and the liver/body weight ratio was lower (Figures 1D, E). We also analysed the liver function markers alanine transaminase (ALT) and aspartate aminotransferase (AST) in serum samples. Our results showed that these two markers were significantly lower in the STA group than in the control group. This suggests that liver function damage can be reversed when STA is administered at high doses (Figure 1F). The findings from gross liver tissue photographs (Figure 1G) and H&E staining results provided additional confirmation of the above results (Figure 1H). In the metastatic model, a dose-dependent reduction in the number and size of tumor nodules and lesion area was observed following STA treatment compared to the control group. Histological analysis of the heart, spleen, lungs, and kidneys in both the high-concentration STA group and the control group did not reveal any significant tissue changes, suggesting that this concentration does not exhibit any apparent toxicity in cancer metastatic mice (Figure 1I).

### 3.2 STA affects the polarisation of tumour associated macrophages in CRLM

**FIGURE 2:**
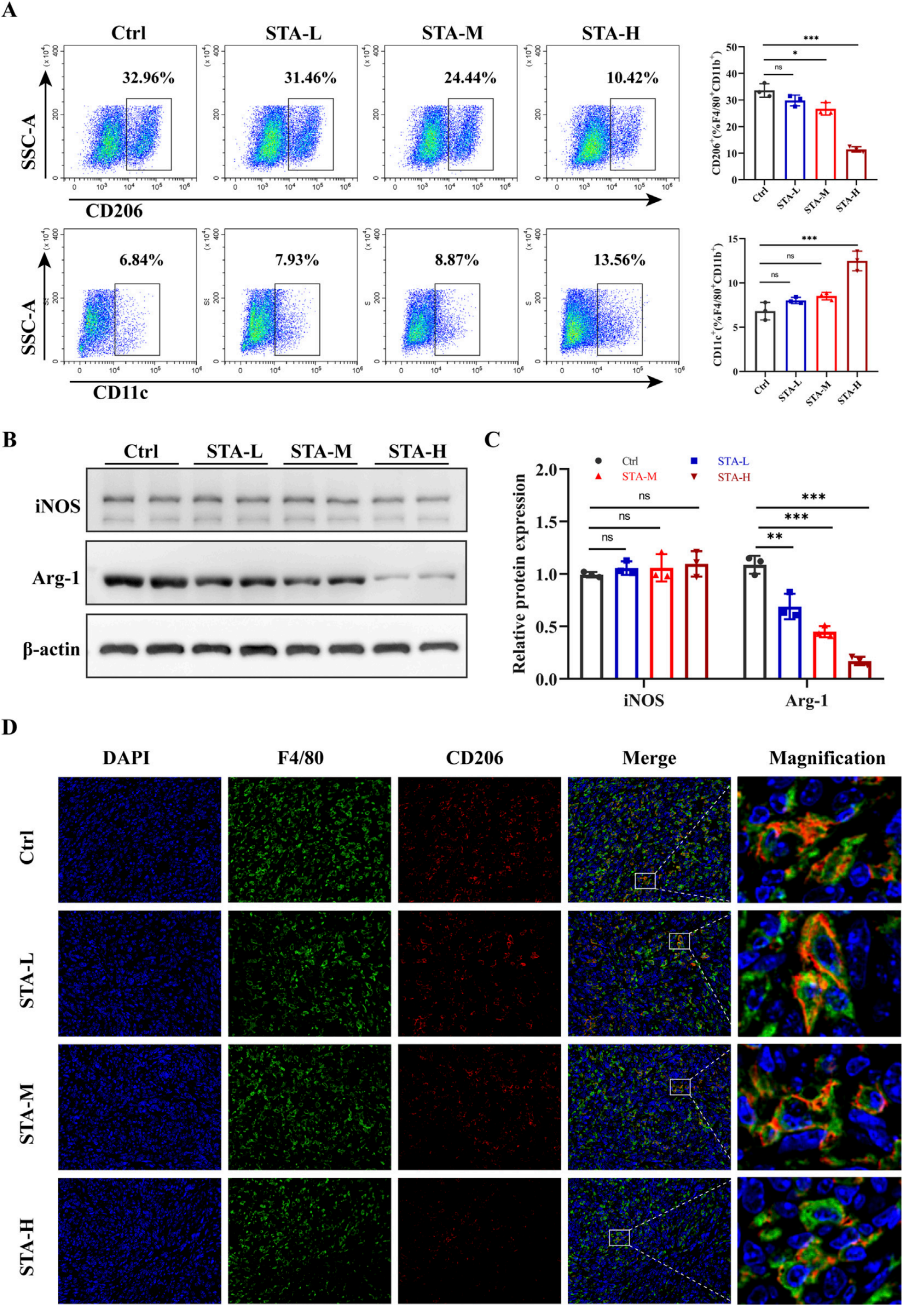
STA inhibits M2 polarization of macrophages in liver metastases. **(A)** Flow cytometry analysis of the ratio of M1 (CD11c^+^) and M2 (CD206^+^) macrophage populations of F4/80^+^/CD11b^+^ TAMs isolated from liver metastases. **(B)** Arg-1 and iNOS were detected by western blotting in mouse liver metastases. **(C)** Quantitative analysis of protein expression. **(D)** Immunofluorescence staining of liver tissues with F4/80^+^(green), CD206^+^(red) and DAPI (blue). Data are shown as the mean ± SD, n = 3–4.

Macrophage polarization has the potential to influence tumor metastasis. In light of these findings, we proceeded to investigate the polarization status of macrophages in liver metastases. Previous studies have categorized TAMs into M1-macrophages and M2-macrophages based on the expression of CD11c and CD206 markers. Flow cytometry demonstrated a reduction in the proportion of M2-TAMs (CD206^+^F4/80^+^CD11b^+^) in liver metastases following treatment with medium-dose and high-dose STA, accompanied by an increase in the proportion of M1- TAMs (CD11c^+^F4/80^+^CD11b^+^) following high-dose STA. In addition, a trend towards an increase in M1- TAMs was observed in the low and medium dose groups, although this was not statistically significant (Figure 2A). Therefore, we speculated that STA exerts its anti-metastatic effect through M2 macrophages. iNOS and Arg-1 are the major markers of M1- TAMs and M2- TAMs, respectively. We wanted to further verify the polarization of macrophages in liver metastases after STA treatment. Western blot was used to detect the protein expression of Arg-1 and iNOS in liver metastases. Notably, the expression of Arg-1, a marker of M2- TAMs, was significantly suppressed in liver metastases after STA treatment (Figures 2B, C). There was no discernible increase in the expression of iNOS protein in M1- TAMs following STA treatment in comparison to the control group. Based on these findings, we hypothesize that STA exerts its effects on macrophage polarization, specifically affecting M2 polarization rather than M1, this causes M1- TAMs ratio is relatively lower. The immunofluorescence results demonstrated that the number of M2- TAMs was significantly higher in the liver metastases of the control group, in contrast to the STA treatment group. It can be posited that STA treatment may influence the proportion of M2-TAMs in liver metastases (Figure 2D). In conclusion, the results demonstrated that STA inhibited the polarization of M2-macrophages in metastases.

### 3.3 The STA-mediated anti-tumor metastatic effect is dependent on macrophages

**FIGURE 3:**
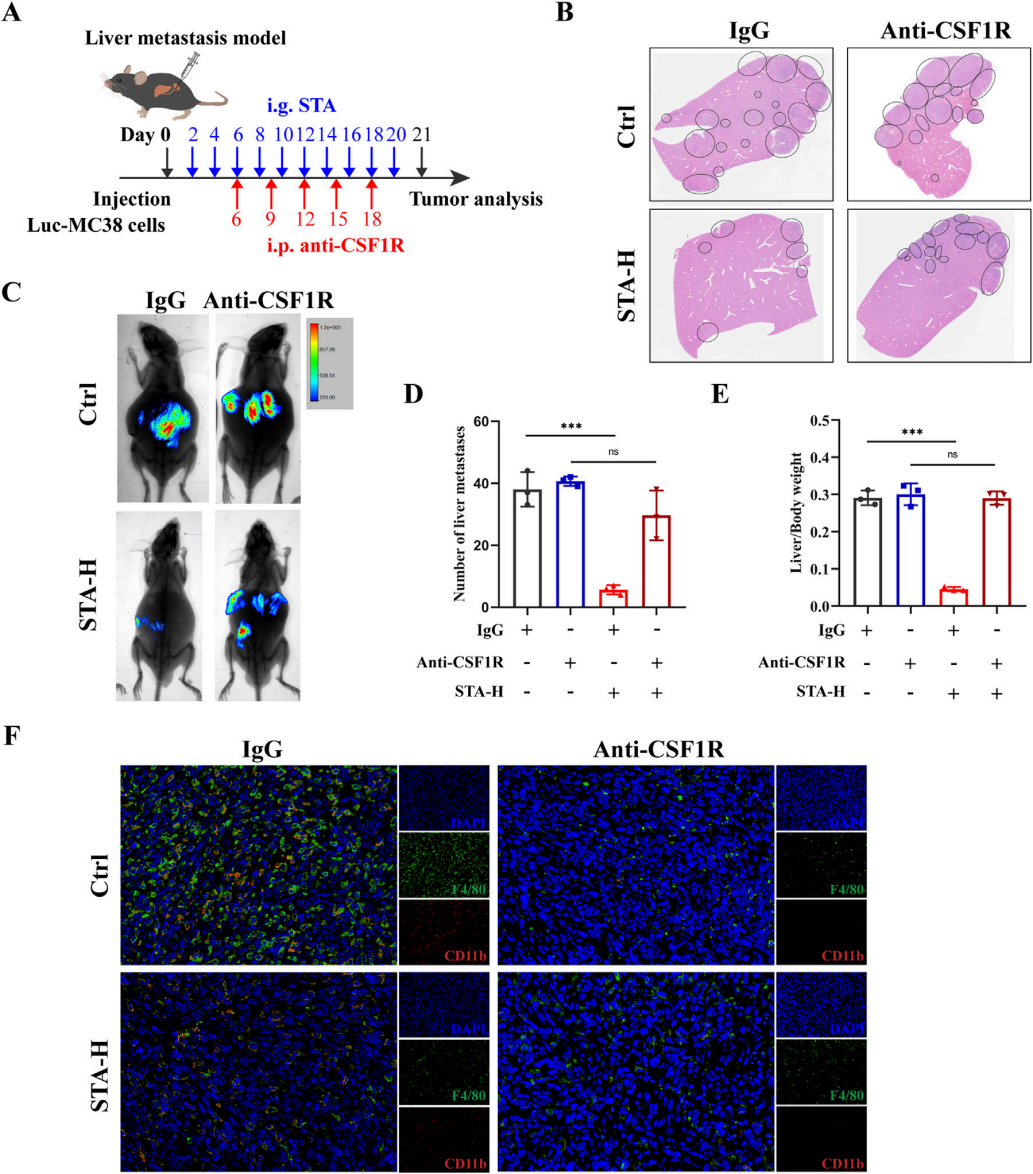
STA inhibits tumor metastasis through macrophages. **(A)** Workflow for STA treatment with or without macrophage depletion; i.p., intraperitoneally. **(B)** Hepatic metastatic foci formation was observed using HE staining, with a scale bar of 5 mm. **(C)** Live imaging was conducted to monitor tumor growth within the mice of each group. **(D)** Number of liver metastases. **(E)** Liver/body weight. **(F)** Immunofluorescence staining of liver tissues with F4/80^+^ (green), CD11b^+^ (red) and DAPI (blue). Data are shown as the mean ± SD, n = 3–4.

The aforementioned studies have demonstrated that STA inhibits the M2 polarization of TAMs in CRLM. It can be concluded that STA may play an anti-tumor metastasis role by inhibiting the M2 polarization of TAMs. To further substantiate the hypothesis that macrophages are the primary factor responsible for STA’s inhibition of CRLM, we employed an anti-CSF1R monoclonal antibody to deplete macrophages in a mouse liver metastasis model (Figure 3A). The results demonstrated that STA-H exhibited a pronounced inhibitory effect on the occurrence of liver metastasis of colon cancer in the presence of macrophages. However, following the removal of macrophages, the number and size of liver metastases in the STA-H group were not significantly different from those in the control group. This indicates that the removal of macrophages significantly reduced the ability of STA to inhibit the formation of CRLM (Figures 3B–E). The results of immunofluorescence indicated that the expression of TAMs in the liver metastases of mice was significantly inhibited following the administration of anti-CSF1R compared to the IgG group (Figure 3F). This suggests that STA exerts its anti-tumor metastatic effect by inhibiting macrophage M2 polarization, rather than tumor-associated macrophage recruitment.

### 3.4 STA inhibits M2-like macrophage polarization *in vitro*

**FIGURE 4:**
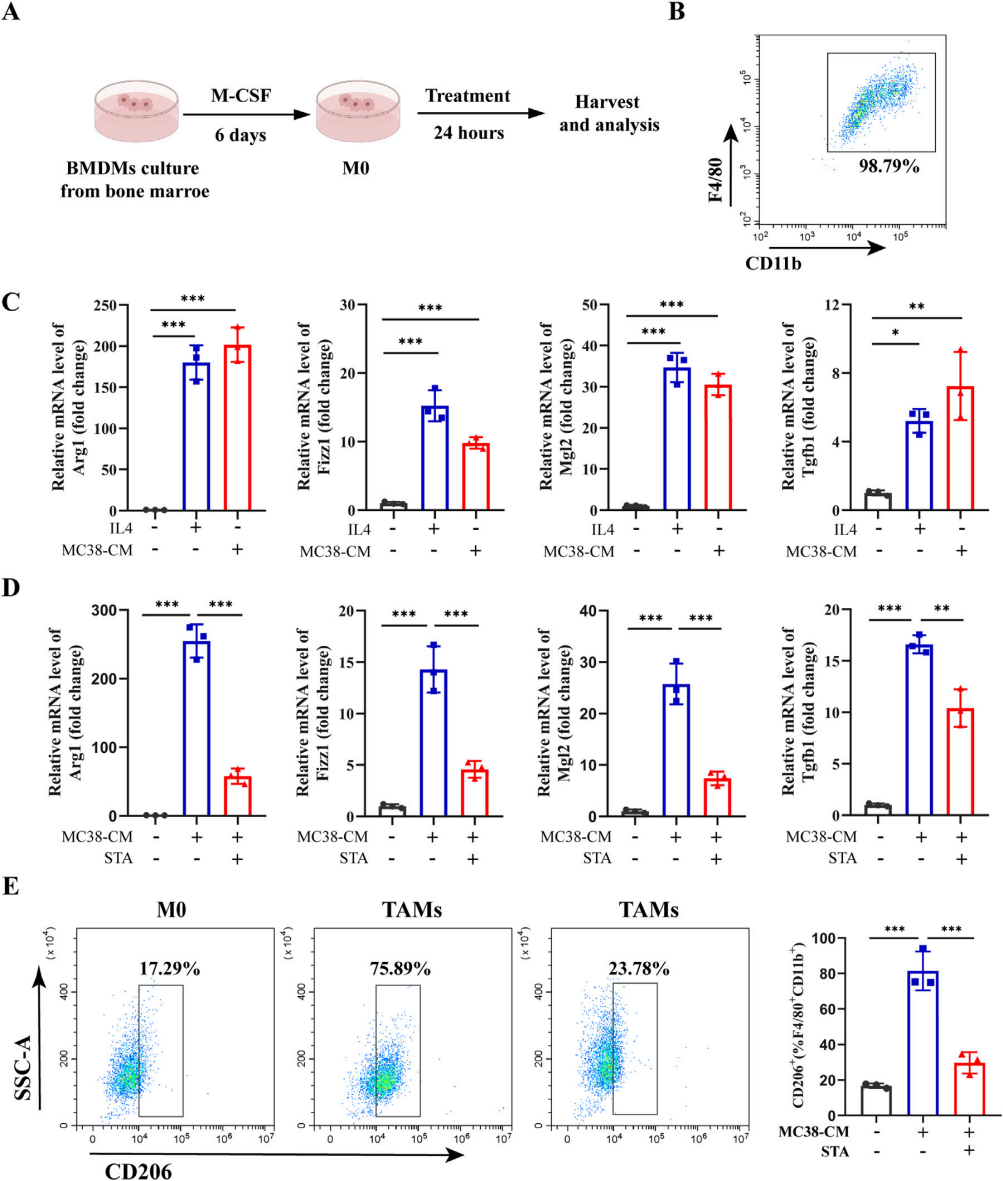
STA inhibits M2 polarization of tumor-associated macrophages *in vitro*. **(A)** Bone marrow cells were extracted from C57BL/6 mice and induced with M-CSF to differentiate into M0 macrophages *in vitro*. **(B)** Flow cytometry was utilized to validate the differentiation of BMDMs into mature M0 macrophages by surface markers F4/80 and CD11b. **(C)** BMDM were isolated from WT mice and induced with IL-4 to generate M2-like macrophages, or induced with MC38-CM to generate tumor-associated macrophages, the expression of M2 markers (Arg1, Fizz1, Mgl2, and Tgfb1) were measured by qRT-PCR. **(D)** BMDM were isolated from WT mice and induced with MC38-CM to generate TAMs, followed by treatment with or without STA, the expression of M2 markers (Arg1, Fizz1, Mgl2, and Tgfb1) were measured by qRT-PCR. **(E)** Flow cytometry analysis was conducted to assess the proportion of M2 macrophages (CD206+) in TAMs treated with or without STA. Data are shown as the mean ± SD, n = 3–5. *p < 0.05, **p < 0.01, and ***p < 0.001.

To explore the potential mechanism by which STA inhibits CRLM via macrophages, BMDMs were isolated from wild-type mice (Figure 4A) and induced to differentiate into mature M0 macrophages as evidenced by expression of the surface markers F4/80 and CD11b (Figure 4B). In order to more accurately reflect the *in vivo* tumor environment, we utilize MC38-conditioned medium (MC38-CM) processed macrophages. BMDMs were treated with MC38-CM to induce M0 macrophages to construct the TAMs phenotype, and IL-4 was used to induce M0 macrophages to differentiate into the M2 phenotype. The expression of M2 polarization markers (Fizz1, Mgl2, Arg1, and Tgfb1) in M2- TAMs and TAMs was markedly elevated in comparison to M0 (Figure 4C). Following the induction of M2 polarization of macrophages by MC38-CM, we observed that STA treatment resulted in the suppression of M2 marker expression, a finding that was confirmed by qRT-PCR (Figure 4D). Furthermore, flow cytometric analysis of cell surface markers confirmed the polarization of these macrophages. The proportion of CD206^+^ macrophages (% F4/80^+^CD11c^+^) in TAMs group increased from 17.29% to 75.89%. However, following STA treatment, the proportion of CD206^+^macrophages (%/F4/80^+^CD11c^+^) decreased to 23.78%, indicating that STA significantly inhibited the M2 polarization of TAMs *in vitro* (Figure 4E). This result demonstrated that STA treatment suppressed the expression of M2 markers in TAMs.

### 3.5 STA suppress the metastatic potential of CRC cells dependent on the presence of M2 macrophages

**FIGURE 5:**
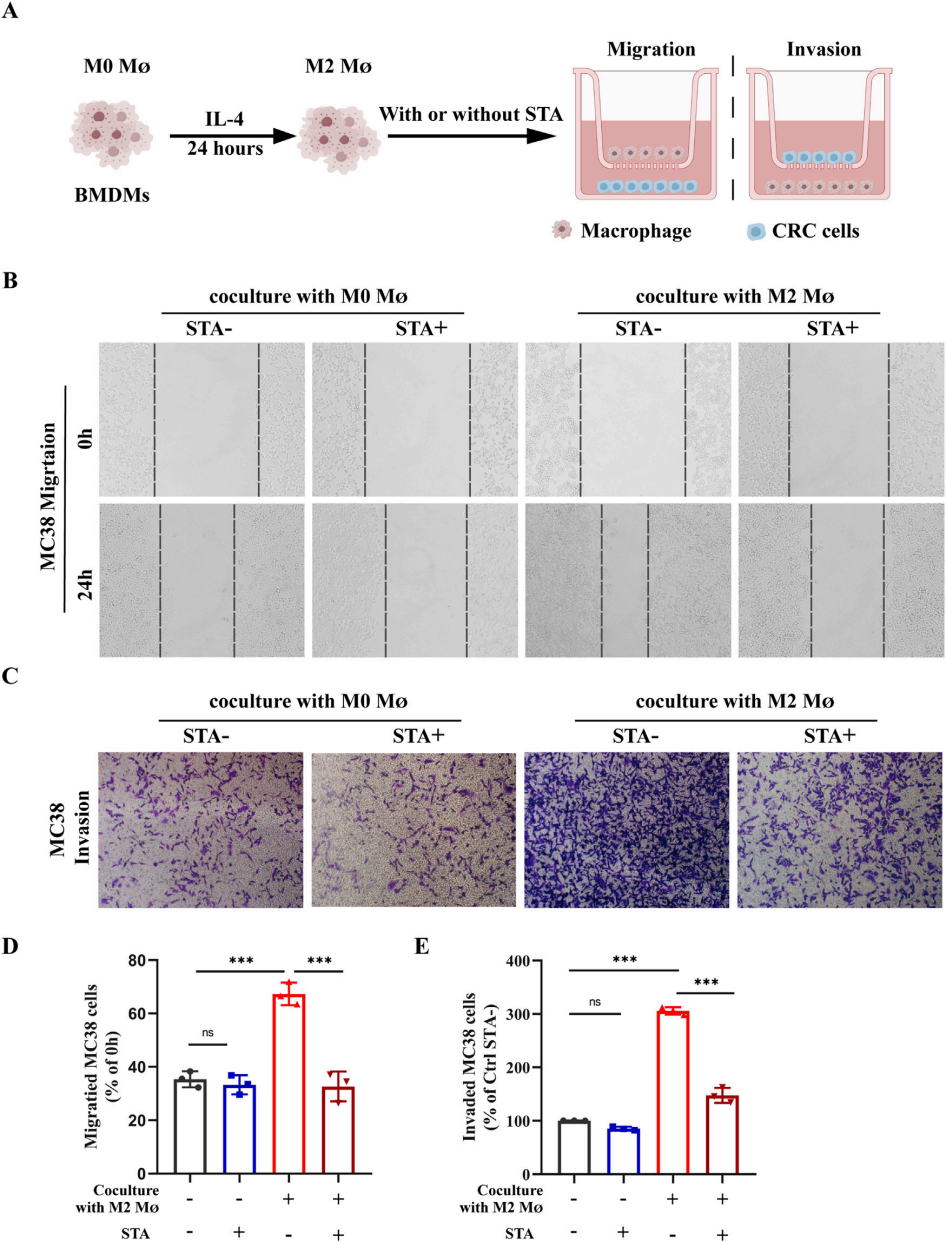
STA suppress the metastatic potential of CRC cells in a manner that is dependent on the presence of M2 macrophages. **(A)** Schematic representation of experimental conditions; MØ: macrophage **(B)** Scratch assay was used to measure MC38 cell migration. **(C)** Transwell assay was employed to assess MC38 cell invasion. **(D)** Quantitative analysis of MC38 cell migration. **(E)** Quantitative analysis of MC38 cell invasion. Data are shown as the mean ± SD, n = 3–4.

The ability of cancer cells to migrate is of great importance in the context of cancer metastasis. To investigate the effect of M2-TAMs in the TME on CRC cells migration and the role played by STA, an *in vitro* induction of M2- TAMs was conducted. The control group was comprised of MC38 cells and M0 macrophages, which were co-cultured with or without STA treatment. The experimental group was conducted under the cell culture conditions shown in Figure 5A. MC38 cells and M2-TAMs were co-cultured on Transwell cell culture plates for 24 h, and the co-culture system was treated with 10 μM STA. The impact of this treatment on CRC cell migration and invasion was then evaluated. The results demonstrated that the co-culture of MC38 cells and M2 macrophages for 24 h significantly enhanced the capacity of CRC cells to migrate, with an increase from 32.3% to 71.9%. It is notable that in the control group, CRC cells exhibited a migration rate of 32.3% independently. In the control group, the STA process was unable to inhibit CRC cells migration. In the co-culture M2 macrophages system, however, the STA significantly reduced CRC cells migration, suggesting that the STA inhibits CRC migration by regulating M2 macrophages (Figures 5B, D). The invasion assay results demonstrated that the co-culture of M2 macrophages enhanced the invasion ability of CRC cells. Conversely, STA significantly inhibited the invasion ability of CRC cells in the M2 macrophages co-culture system, reducing the migration ability from 298.5% to 145.6% (Figures 5C, E). These data indicate that STA can inhibit the migration and invasion of CRC cells induced by M2 macrophages.

### 3.6 STA inhibition of tumor angiogenesis through M2 macrophages

**FIGURE 6:**
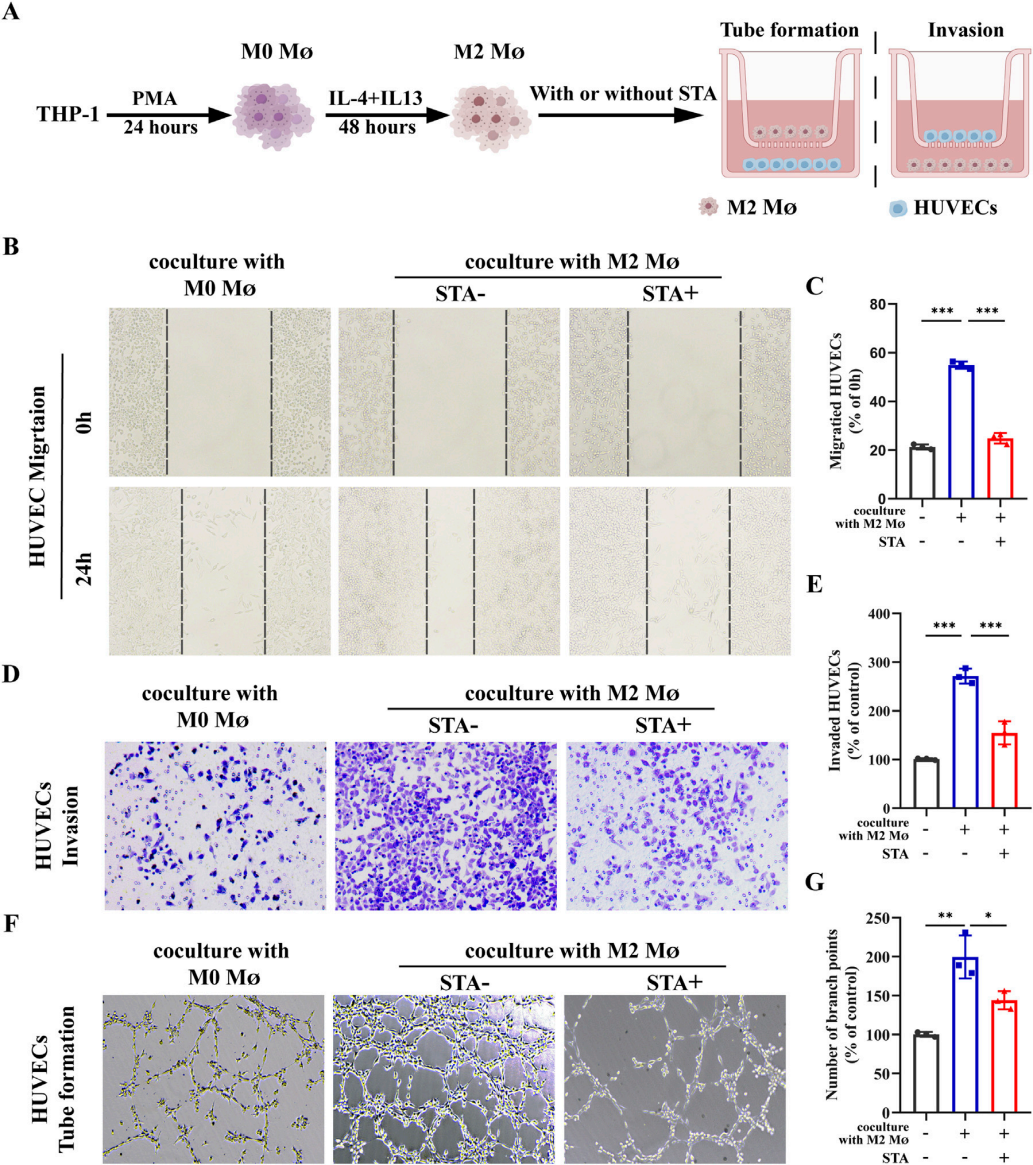
STA suppresses M2 macrophages-induced tumor angiogenesis. **(A)** Schematic representation of experimental conditions. **(B)** Scratch assay was used to measure HUVECs migration. **(C)** Quantitative analysis of HUVECs migration. **(D)** Invasion ability of HUVECs was assessed using the Transwell assay. **(E)** Quantitative analysis of HUVECs cell invasion. **(F)** Tube formation assay was conducted to evaluate the tube-forming ability of HUVECs. **(G)** Quantitative analysis of tube formation in HUVECs. Data are shown as the mean ± SD, n = 3–4.

Angiogenesis is a necessary condition for cancer cells to transport nutrients and exclude metabolites. It is an important part of tumor progression and plays a key role in tumor growth and metastasis. Inhibition of angiogenesis can be used as a therapeutic strategy for tumor therapy. Endothelial cell migration is one of the hallmarks of angiogenesis and one of the early steps in the angiogenic cascade. The control group was comprised of M0 macrophages co-cultured with HUVECs, while the experimental group consisted of HUVECs and M2 macrophages co-cultured on Transwell cell culture plates for 24 h with or without 10 μM STA treatment of the co-culture system (Figure 6A). The subsequent evaluation of HUVECs migration and invasion was conducted. The results demonstrated that in comparison to the M0 macrophage, the M2 macrophages cultured HUVECs exhibited enhanced migratory capacity. The STA-treated HUVECs exhibited a reduction in migratory capacity, with the percentage of cells migrating reduced from 55.13% to 25.68% (Figures 6B, C). Similarly, co-culture with M2 macrophages increased the cell invasion ability of HUVECs, and STA treatment effectively reduced the effect of M2 macrophages on the invasion ability of HUVECs (Figures 6D, E). Furthermore, the influence of M2 macrophages on vascular formation was evaluated in STA HUVECs. The impact of HUVECs incubated for 6 h in the matrix was also assessed, as was the structure of the matrix surface gel capillary sample. The M2 macrophages were then trained, resulting in an increase of branch points by 179%. However, after processing, this effect was suppressed to 143% (Figures 6F, G). These findings suggest that STA can inhibit M2 macrophage-induced angiogenesis.

### 3.7 Involvement of JAK2/STAT3 signaling in STA-mediated inhibition of M2 macrophage polarization

**FIGURE 7:**
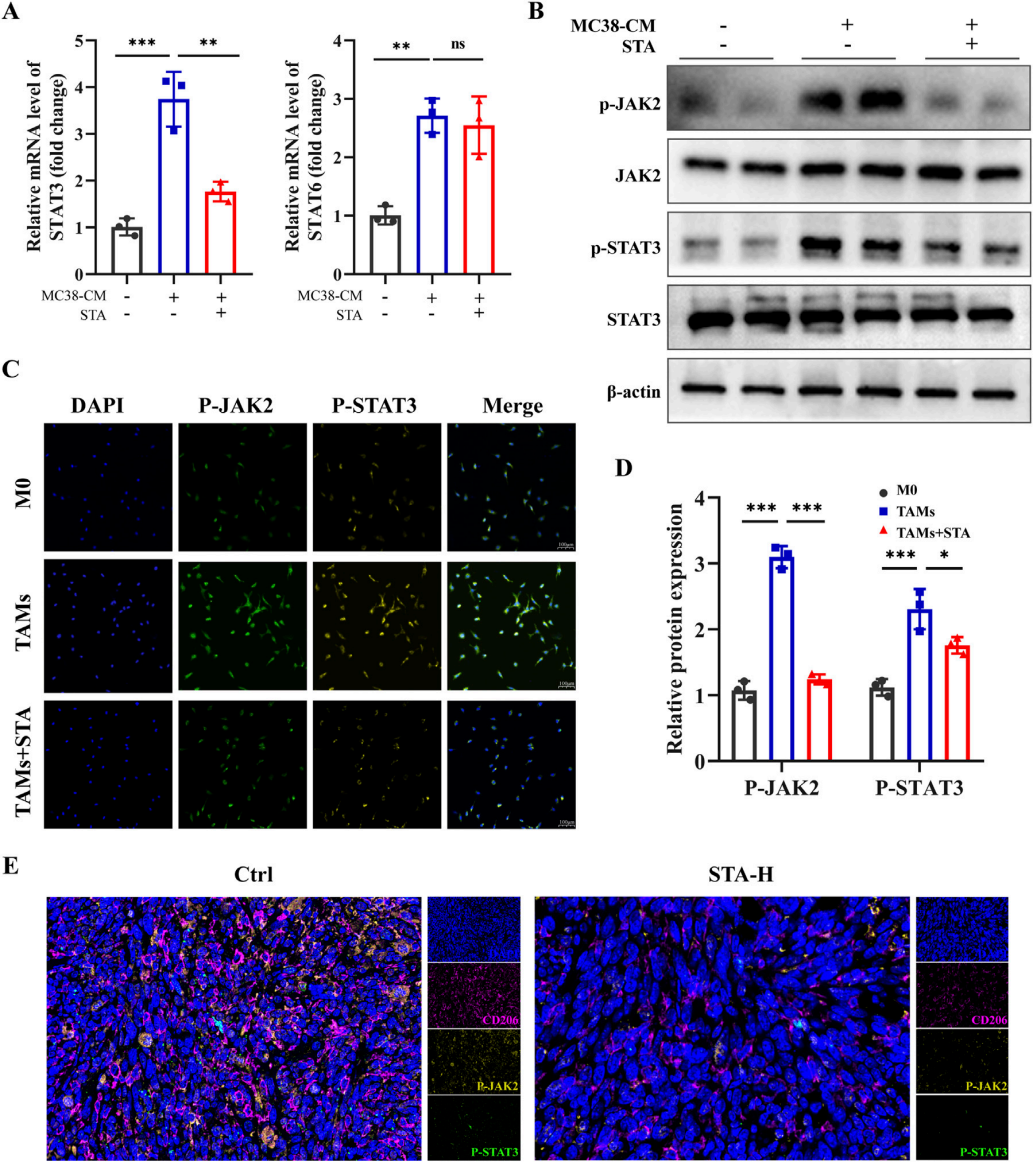
JAK2/STAT3 signaling pathway is involved in STA inhibiting M2 polarization of macrophages. **(A)** The expression levels of STAT3 and STAT6 genes in tumor-associated macrophages after STA treatment were assessed using qRT-PCR. **(B)** Western blot analysis was performed to evaluate the protein expression of p-JAK2, JAK2, p-STAT3, and STAT3 in tumor-associated macrophages after STA treatment. **(C)** Immunofluorescent staining with p-JAK2, p-STAT3, and DAPI was conducted on macrophages treated with different conditions. **(D)** Quantitative analysis of p-JAK2, JAK, p-STAT3, and STAT3 protein expression in each group was performed. **(E)** Immunofluorescence staining of liver tissues with CD206, p-JAK2, p-STAT3, and DAPI. Scale bar: 100 μm. Data are shown as the mean ± SD, n = 3.

We further investigated the specific mechanism by which STA inhibits macrophage M2 polarization. Previous studies have shown that the STAT3 and STAT6 pathways are central to macrophage M2 polarization (Deng et al., 2024; Su et al., 2024). Using qRT-PCR, we observed that the expression levels of STAT3 and STAT6 were upregulated in macrophages exposed to MC38-CM compared to the control group. However, after treatment with STA, the expression of STAT3 decreased, while there was no significant change in the level of STAT6 expression (Figure 7A). Given that activation of JAKs facilitates the phosphorylation of members of the signal transducer and activator of the transcription (STAT) family, our study sought to assess the expression of JAK2 and STAT3 in macrophages treated with STA using Western blotting. The results showed a significant reduction in the phosphorylation levels of JAK2 and STAT3 in TAMs treated with STA (Figures 7B, D). As shown in Figure 7C, the levels of p-STAT3 and P-AKT2 in macrophages increased after treatment with MC38-CM, whereas the levels of p-STAT3 in macrophages decreased after treatment with MC38-CM and STA. Consistent with the *in vitro* results, in the mouse model of liver metastasis, multicolor immunofluorescence results showed that the levels of p-STAT3 and P-AKT2 in the liver of STA-H mice were decreased compared with those in the control group (Figure 7E). Taken together, these results demonstrate the importance of JAK2/STAT3 signaling in regulating the inhibitory process of STA-mediated macrophage M2 polarization.

### 3.8 Activation of the JAK2/STAT3 pathway reversed STA’s ability to inhibit CRC metastasis and angiogenesis

**FIGURE 8:**
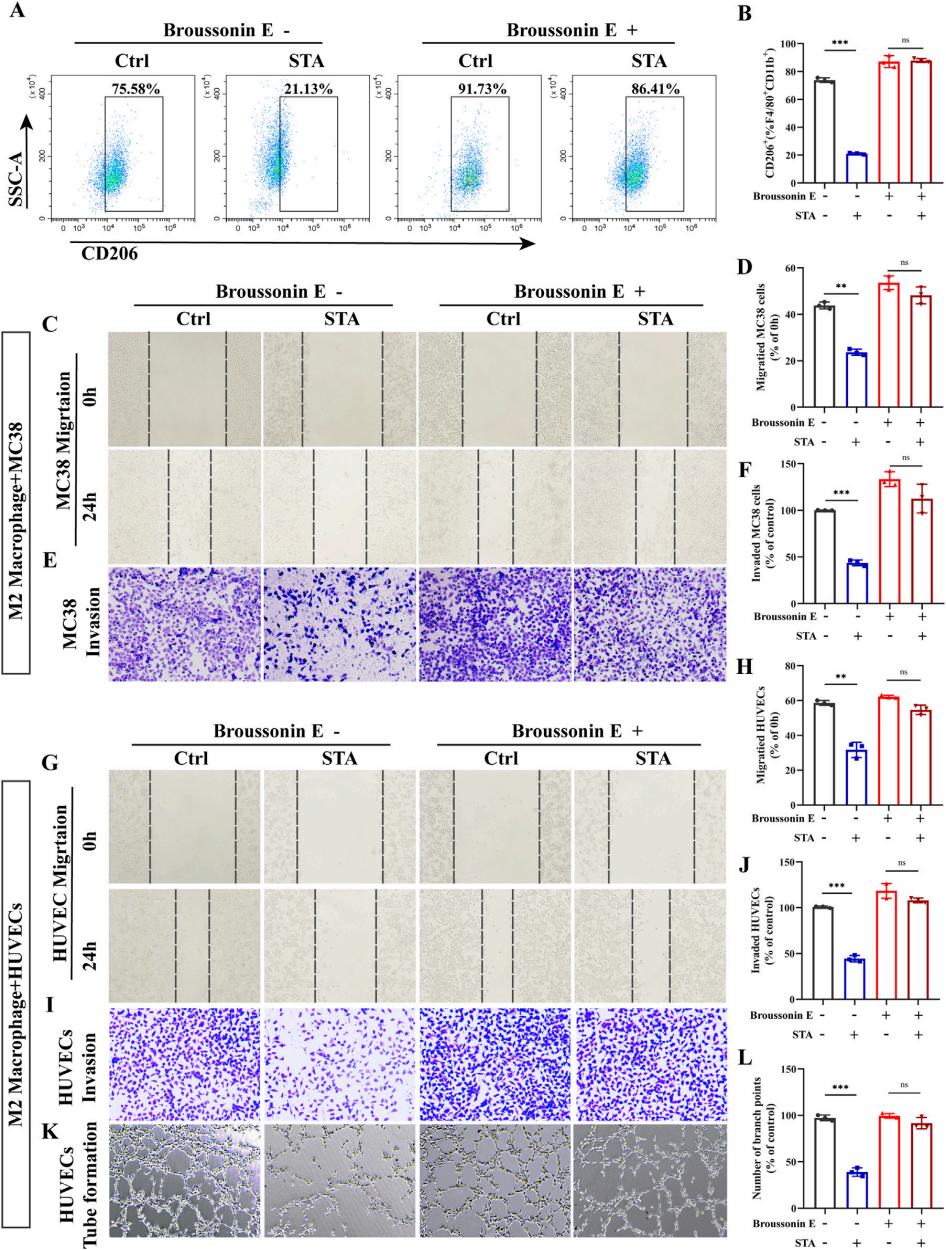
Activation of the JAK2/STAT3 pathway reversed the ability of STA to inhibit colorectal cancer metastasis and angiogenesis. **(A)** Tumor-associated macrophages were incubated with or without Broussonin E in the presence of STA for 48 h, and flow cytometry was used to detect the expression of M2 macrophages. **(B)** Graphical representation of the proportion of M2 macrophages. **(C)** Scratch assay was used to measure MC38 cell migration. **(D)** Quantitative analysis of MC38 cell migration. **(E)** Transwell assay was employed to assess MC38 cell invasion. **(F)** Quantitative analysis of MC38 cell invasion. **(G)** Scratch assay was used to measure HUVECs migration. **(H)** Quantitative analysis of HUVECs migration. **(I)** Invasion ability of HUVECs was assessed using the Transwell assay. **(J)** Quantitative analysis of HUVECs cell invasion. **(K)** Tube formation assay was conducted to evaluate the tube-forming ability of HUVECs. **(L)** Quantitative analysis of tube formation in HUVECs. Data are shown as the mean ± SD, n = 3.

Since JAK2/STAT3 may play a critical role in macrophage M2 polarization, we used the JAK2/STAT3 activator Broussonin E to confirm that JAK2/STAT3 signaling is involved in STA inhibiting the M2 polarization of macrophages. Surprisingly, after Broussonin E treatment, the inhibitory effect of STA on macrophage M2 polarization was reversed (Figures 8A, B). The objective of this study was to investigate the effect of STA on M2- TAMs in the TME and their role in regulating CRC cell migration following JAK2/STAT3 activation. To this end, IL4-induced M2 macrophages were co-cultured with MC38 cells, and the ability of CRC cells to migrate and invade was then assessed. The results of the scratch assay indicated that the inhibitory effect of STA on CRC cell migration and invasion through M2 macrophages was diminished when JAK2/STAT3 signaling was activated (Figures 8C–F). Then, M2 macrophages were induced by IL4 and IL13 and co-cultured with HUVECs to assess their migration, invasion, and angiogenesis abilities. The results demonstrated that the inhibitory effect of STA on HUVEC migration, invasion, and angiogenesis via M2 macrophages was attenuated when JAK2/STAT3 signaling was activated in comparison to the control (Figures 8G–L).

### 3.9 STA promotes the infiltration of CD8^+^T cells in TME by reducing the infiltration of M2 cells in TME and enhances the anti-tumor efficacy of anti-PD-1

**FIGURE 9:**
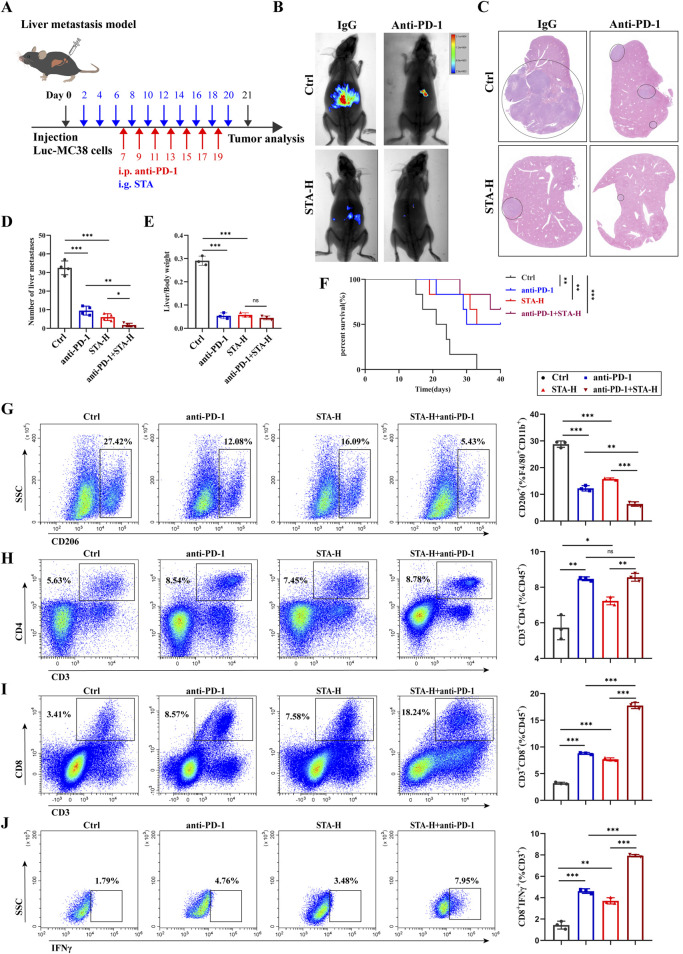
STA combined with PD-1 monoclonal antibody can effectively inhibit the occurrence and development of liver metastasis. **(A)** Experimental protocol for combination therapy. **(B)** Live imaging was performed to monitor tumor growth within the mice of each group. **(C)** Hepatic metastatic foci formation was observed using HE staining, with a scale bar of 5 mm. **(D)** Number of liver metastases. **(E)** Liver/body weight. **(F)** Kaplan–Meier survival curves were plotted for mice in the Ctrl, anti-PD-1, STA-H, and anti-PD-1+STA-H groups (n = 6). **(G)** Representative flow cytometry results and quantification of the proportion of M2 macrophages in hepatic metastases of mice treated with control, anti-PD-1, STA-H, and combined anti-PD-1 with STA therapy. **(H)** Representative flow cytometry results and quantification of the proportion of CD4^+^ T cells in hepatic metastases of mice treated with control, anti-PD-1, STA-H, and combined anti-PD-1 with STA therapy. **(I)** Representative flow cytometry results and quantification of the proportion of CD8^+^ T cells in hepatic metastases of mice treated with control, anti-PD-1, STA-H, and combined anti-PD-1 with STA therapy. **(J)** Representative flow cytometry results and quantification of the proportion of CD8^+^ T IFNγ^+^ cells in hepatic metastases of mice treated with control, anti-PD-1, STA-H, and combined anti-PD-1 with STA therapy. Data are shown as the mean ± SD, n = 3.

The research studies discussed in this paper have shown that STA has the potential to mitigate the primary factor driving the CRLM, specifically the immunosuppressive tumor microenvironment. Therefore, it is anticipated that STA therapy could counteract immune evasion mechanisms and effectively target liver metastases. Furthermore, liver metastases exhibit limited responsiveness to immunotherapy, which constrains the effectiveness of anti-PD-1 treatment in managing CRLM. A model of colorectal cancer liver metastasis was established and STA was co-administered with anti-PD-1 (Figure 9A). In comparison to mice treated solely with either anti-PD-1 or STA, those receiving a combined treatment of anti-PD-1 and STA exhibited significantly restricted tumor growth, with the observed differences being statistically significant. After combination therapy, liver metastasis of CRC was almost completely inhibited (Figures 9B, C). In addition, the number of liver metastases was significantly reduced in the anti-PD-1 and STA groups compared with the control group, and the combination group had a better effect (Figure 9D). The ratio of liver/body weight was also significantly reduced after anti-PD-1 and STA treatment (Figure 9E). Moreover, we observed that the combination treatment improved the survival rate of mice with liver metastases (Figure 9F). These results indicate that the combination of STA and PD-1 showed the most significant inhibition of the growth of CRLM in mice. Qualitative and quantitative analysis of immune cell proportions and functions within liver metastatic lesions was also performed using flow cytometry. STA combined with anti-PD-1 therapy inhibited the proportion of M2 macrophages in metastatic lesions, with superior efficacy compared to monotherapy (Figure 9G). Both STA and anti-PD-1 treatment increased the proportion of CD4^+^ T cells within tumors compared to the control group, but the combination therapy showed no difference in efficacy compared to anti-PD-1 monotherapy (Figure 9H). In addition, both STA and anti-PD-1 treatments increased the proportion of CD8^+^ T cells within tumors, with the combination of STA and anti-PD-1 showing the greatest increase in CD8^+^ T cell proportion, indicating enhanced immunotherapeutic effects (Figure 9I). IFN-γ plays a pivotal role in T cell-mediated tumor cell control. The most pronounced increase in the proportion of CD8^+^ IFN-γ^+^ T cells within the tumor was observed following combination therapy, suggesting that STA enhanced cytotoxic T cell activity (Figure 9J). In conclusion, the results indicate that STA alters the immune microenvironment from immunosuppression to immune activation. Furthermore, the combined use of STA and anti-PD-1 has been shown to have synergistic therapeutic effects, enhancing the efficacy of immunotherapy and inhibiting liver metastasis in CRC.

## 4 Discussion

In the process of discovering and developing novel anti-cancer drugs, STA emerges as a promising candidate. Extensive research has been conducted on STA’s anti-cancer activity, with its main mechanisms including inhibition of cancer cell proliferation (Zeng et al., 2023), induction of cell cycle arrest and apoptosis in cancer cells (Zhai et al., 2024), and induction of autophagy and promotion of cell senescence (Bao et al., 2022). However, the anti-tumor metastasis and immune modulation effects of STA have long been overlooked. Therefore, this study focuses on exploring the potential role of STA in inhibiting CRLM through immune regulation. By investigating the progression of CRLM, *in vitro* and *in vivo* tumor growth induced by TAMs M2 polarization, angiogenesis, and metastasis, this study examines the anti-tumor metastasis efficacy of STA in CRLM. Importantly, the potential anti-tumor mechanisms of STA confirm that the JAK2/STAT3 pathway in macrophages is a potential therapeutic target.

More than 50% of CRC patients will develop liver metastasis, which is a major cause of clinical treatment failure and late-stage patient death, severely constraining the survival and prognosis of CRC patients (Peng et al., 2021). The median survival time for untreated patients with CRLM is typically 5–10 months. However, with effective treatment, the 5-year survival rate can increase to 20%–40% and the median survival time can be extended to 28–40 months (Rauchfuß et al., 2019). Consequently, patients with CRLM should adopt a proactive approach towards treatment. However, treating tumor metastasis is a major challenge in clinical practice. Tumor metastasis is an extremely complex process involving different pathological steps, including immune escape of tumor cells from the primary site, survival in the circulatory system, dissemination to other organs, and distant colonization (Li et al., 2021). Increasing evidence suggests that TAMs in malignant tumors often exhibit an M2-like phenotype, exerting immunosuppressive effects that promote tumor development, stimulate tumor angiogenesis, and enhance tumor migration and invasion, ultimately leading to tumor metastasis (Liu et al., 2023; Zhan et al., 2023; Chen et al., 2022). Consequently, the targeting of M2-type TAMs may be regarded as a promising and efficacious strategy for the control of tumor metastasis.

This study represents the inaugural demonstration of the inhibitory effect of STA on the progression of CRLM in mice. The results demonstrated that STA treatment for 21 days resulted in a dose-dependent reduction in the number and volume of liver metastases, as well as an alleviation of liver dysfunction in mice with CRC. The administration of a high dose (60 mg/kg) of STA was found to extend the survival period of mice without inducing any toxic effects. Moreover, we focused on investigating the regulatory effect of STA on TAMs M2 polarization. Flow cytometry results showed a decrease in the proportion of M2 TAMs in liver metastases, which was consistent with immunofluorescence results and Western blot results. Interestingly, the ability of STA to inhibit liver metastasis was offset after we cleared macrophages from the mice, suggesting that the ability of STA to inhibit liver metastasis is achieved through the regulation of macrophages. Based on this, we isolated BMDMs from mice and differentiated them into mature M0 macrophages. Then treated M0 macrophages with MC38-CM to induce TAMs *in vitro* and evaluated the polarization spectrum of TAMs induced by tumor cell-conditioned medium using defined macrophage polarization state genes, the results confirming the M2 polarization of TAMs in the CRLM tumor microenvironment and STA inhibits the M2 polarization of TAMs *in vitro*. Furthermore, we further confirmed STA can effectively inhibit MC38 cells migration and invasion by inhibiting macrophage M2 polarization, as well as inhibit angiogenesis, which is a key process in tumor metastasis spread.

A previous study identified the JAK2/STAT3 signal as a key driver of macrophage activation in gastric tumors. This activation process resulted in the transformation of macrophages into M2 subtypes of tumor-associated macrophages (Akad et al., 2023). This study investigated the role of the JAK2/STAT3 signaling pathway in STA-mediated inhibition of TAMs M2 polarization. The results of the study indicated that the JAK2/STAT3 pathway was activated in TAMs following co-culture of mc38 conditioned medium. However, the phosphorylation status of JAK2 and STAT3 in the TAMs was reduced following STA treatment. Additionally, we utilized Broussonin E, an activator of the JAK2/STAT3 pathway, to substantiate the involvement of STA in the M2 polarization of macrophages through JAK2/STAT3 signaling. In the absence of Broussonin E treatment, STA significantly reduced the increase in CD206-positive macrophages induced by MC-38-CM. However, after Broussonin E treatment, the inhibitory effect of STA on macrophage M2 polarization was offset. It is noteworthy that when M2 macrophages were co-cultured with MC38 cells, the efficacy of STA in inhibiting tumor cell migration and angiogenesis was diminished following treatment with Broussonin E. This observation corroborates that STA exerts an anti-tumor effect by impeding TAMs M2 polarization through the inhibition of the JAK2/STAT3 signaling pathway.

With technological advancements, ICB therapy has emerged as a significant treatment modality for tumor metastasis (Wang et al., 2022). These antibodies target regulatory pathways in T cells, enhancing anti-tumor immune responses, leading to significant clinical advancements, and offering a new anti-cancer strategy (Liu et al., 2021). However, as the clinical application of immune checkpoint inhibitors deepens, some studies have observed that some patients initially do not respond, which is closely related to the heterogeneity of the TME (Kawashima et al., 2021). Therefore, overcoming immune-suppressive microenvironments holds promise as a breakthrough solution for preventing and treating CRC liver metastasis, with significant implications for further extending patient survival. Studies have found that in patients with advanced melanoma, the presence of liver metastases is associated with a decreased proportion and functional suppression of CD8^+^ T cell infiltration compared to other metastatic sites. Previous research suggests that tumor-infiltrating CD8^+^ T cells are predictive factors for the efficacy of ICB therapy such as PD-1 blockade. This study found that STA could synergistically inhibit the formation of CRLM by reducing the infiltration of immune-suppressive macrophages, suppressing tumor-associated macrophage M2 polarization, up-regulating the proportion and activation of CD8^+^ T cell infiltration, and interacting with anti-PD-1 antibodies.

STA exhibits favorable anticancer effects. However, it should not be overlooked that there is a long way to go from animal research to clinical application. The foremost issue that needs to be faced is the relatively poor pharmacokinetic properties of STA, which restricts its clinical application. Research has confirmed that after intragastric administration of stachydrine in rats, the pharmacokinetic results in plasma samples suggest that most of the stachydrine can be rapidly absorbed, quickly metabolized, and directly excreted (Cheng et al., 2020). In response to this problem, researchers are actively exploring solutions. Some studies have synthesized and characterized a series of novel prodrugs of stachydrine. Among them, SS-12 exhibits a longer retention time and higher bioavailability after oral administration in rats, enhancing the hydrophobicity and *in vitro* anticancer activity of STA (Zeng et al., 2023). These studies also encourage us to develop new compounds that are more effective and safer and actively explore the drug development and application of STA. Additionally, regarding the issue that the low bioavailability of natural products limits their application in diseases, some studies have verified that their effects are closely related to the gut microbiota. Targeting the gut microbiota with natural products is one of the breakthrough strategies for treating gastrointestinal diseases (Duan et al., 2024). It has been demonstrated that the active ingredient echinacoside in traditional Chinese medicine shows the efficacy of oral anti-colorectal cancer liver metastasis by promoting butyrate-producing gut bacteria and reducing PI3K/AKT signaling and epithelial-mesenchymal transition (Wei et al., 2024). Regrettably, there are currently no reports on the role of STA in regulating the gut microbiota in tumors. Perhaps this is also an inspiration for us. In-depth analysis of the relationships among STA, the gut microbiota, CRC, and its liver metastasis will surely provide new insights into the treatment mechanism of CRLM and is worthy of further exploration.

## 5 Conclusion

In conclusion, our study revealed that STA-induced inhibition of the JAK2/STAT3 pathway in macrophages can suppress TAMs M2 polarization and further inhibiting tumor cell migration and angiogenesis. Furthermore, STA has the potential to reverse the immunosuppressive microenvironment of liver metastases, increase the proportion of CD8^+^ IFN-γ^+^ T cells infiltration, and enhance the efficacy of immunotherapy with anti-PD-1. STA has shown potential in CRC treatment, but further research is essential for its clinical application. More studies need to be done to improve the bioavailability of STA and explore the molecular mechanisms or targets of STA to pave the way for the development of a STA-based therapeutic strategy.

## Data Availability

The original contributions presented in the study are included in the article/Supplementary Material, further inquiries can be directed to the corresponding author.
