# Supplementary material for: Stachydrine targeting tumor-associated macrophages inhibit colorectal cancer liver metastasis by regulating the JAK2/STAT3 pathway
Source: Front Pharmacol. 2025 Feb 5;16:1514158. doi: 10.3389/fphar.2025.1514158 (PMC11835834; doi:10.3389/fphar.2025.1514158)
Supplement: Supplementary file 1 [file Table1.docx]

| Supplementary Table 1. Summarizing research findings and comparison | | |
| --- | --- | --- |
| **Research Content** | **Details** | **Comparison with References** |
| Effect of STA on colorectal cancer liver metastasis | 1. In the mouse model, STA can inhibit colorectal cancer liver metastasis, reduce the number and size of liver metastases, decrease the liver/body weight ratio, and improve liver function indicators (such as ALT, AST). b. In different STA treatment groups, the survival time of the high-dose group was significantly prolonged, while there was no significant difference in survival time between the low and medium-dose groups and the control group. c. Histological analysis showed that the high-concentration STA group had no significant histological changes in the heart, spleen, lungs, and kidneys, indicating no obvious toxicity. | Similarities: Similar to the inhibitory effects of STA on different tumors in the studies of Liu et al. (2018), Gu et al. (2022), Zhai et al. (2024), and Bao et al. (2022), all indicating that STA has certain anti-tumor activities. Differences: This study focused on colorectal cancer liver metastasis and clarified the inhibitory effect and dose-response relationship of STA in this specific tumor metastasis model. Other studies targeted different tumor types, such as astrocytoma (Liu et al., 2018), chronic myeloid leukemia (Gu et al., 2022), breast cancer ( Zhai et al., 2024), and liver cancer (Bao et al., 2022), and the specific metastasis-related indicators and model details also varied. |
| Effect of STA on the polarization of tumor-associated macrophages (TAMs) | 1. Flow cytometry showed that after treatment with medium and high doses of STA, the proportion of M2 - TAMs in liver metastases decreased, and the proportion of M1 - TAMs increased in the high-dose group, with an increasing trend but not significant in the low and medium-dose groups. b. Western blot detection showed that the expression of the M2 - TAMs marker Arg - 1 in liver metastases was significantly inhibited after STA treatment, and there was no significant change in the expression of the M1 - TAMs marker iNOS protein. c. Immunofluorescence results showed that the number of M2 - TAMs in liver metastases in the STA treatment group was significantly lower than that in the control group. | **Similarities**: In terms of macrophage regulation, it is consistent with the influence of STA on macrophage function in some previous studies, all involving the regulatory effect of STA on macrophage-related phenotypes or markers. |
| Relationship between STA's anti-metastatic effect and macrophages | a. Macrophage depletion experiments showed that after macrophage removal, there was no significant difference in the number and size of liver metastases in the STA - H group compared with the control group, indicating that STA's anti-metastatic effect depends on macrophages. b. Immunofluorescence results showed that the expression of TAMs in liver metastases after anti - CSF1R treatment was significantly lower than that in the IgG group, indicating that STA exerts its anti-metastatic effect by inhibiting macrophage M2 polarization rather than recruitment. | Differences: This study clearly demonstrated the dependence of STA's anti-metastatic effect on macrophages and the specific mechanism related to macrophage polarization through a series of well-designed experiments, which is different from many previous studies that may not have investigated this relationship in such depth. |
| In vitro experiment on STA inhibiting M2-like macrophage polarization | a. Bone marrow cells were isolated from wild-type mice and induced to differentiate into mature M0 macrophages, and then induced into TAMs with MC38 - CM. After STA treatment, the expression of M2 markers (such as Fizz1, Mgl2, Arg1, Tgfb1) was inhibited. b. Flow cytometry analysis showed that the proportion of CD206 ^+^ macrophages in TAMs decreased significantly after STA treatment, indicating that STA inhibited the M2 polarization of TAMs. | **Similarities**: Similar to the influence of STA on cell phenotypes or markers in other studies, all involving the regulatory effect of STA on the expression of related molecules in cells. |
| Effect of STA on the migration and invasion of CRC cells | a. In vitro co-culture experiments showed that M2 macrophages could enhance the migration and invasion ability of CRC cells, and STA treatment could inhibit the migration and invasion of CRC cells induced by M2 macrophages. b. In the Transwell experiment, when M2 macrophages were co-cultured with MC38 cells, STA significantly reduced the migration and invasion ability of CRC cells. | **Similarities**: Consistent with the results of STA inhibiting tumor cell migration and invasion in the studies of Liu et al. (2018) on astrocytoma , all confirming the inhibitory activity of STA in the process related to tumor cell metastasis. **Differences**: This study targeted CRC cells and studied the effect of STA in a co-culture system with M2 macrophages, while other studies targeted different tumor cell types, and there may be differences in experimental systems and detection methods. |
| Effect of STA on tumor angiogenesis | a. In vitro experiments showed that M2 macrophages could enhance the migration and invasion ability of endothelial cells (HUVECs) and promote angiogenesis, and STA treatment could inhibit these effects. b. In scratch assays, Transwell assays, and tube formation assays, the migration, invasion, and tube formation abilities of HUVECs were significantly reduced after STA treatment. | **Similarities**: Consistent with the influence of STA on the process related to tumor angiogenesis explored in some studies, all emphasizing the multi-target effect of STA in tumor microenvironment regulation, involving angiogenesis-related cells in the tumor microenvironment. **Differences**: This study specifically studied the effect of STA on the angiogenesis-related functions of endothelial cells induced by M2 macrophages, and other studies may study the effect of STA on tumor angiogenesis from different perspectives or cell models. |
| Role of the JAK2/STAT3 signaling pathway in STA inhibiting M2 macrophage polarization | a. qRT - PCR results showed that the expression of STAT3 in macrophages decreased after STA treatment, and there was no significant change in the expression of STAT6. b. Western blotting detection showed that the phosphorylation levels of JAK2 and STAT3 in macrophages decreased significantly after STA treatment. c. Immunofluorescence results of the mouse liver metastasis model showed that the levels of p - STAT3 and P - AKT2 in the liver of the STA - H group decreased. | **Similarities**: In terms of signal pathway regulation, it is related to the inhibitory effect of STA on the Akt/Erk and other pathways in the study of Liu et al. (2018), all involving the regulation of intracellular signal pathways by STA. **Differences**: This study clarified the key role of the JAK2/STAT3 signaling pathway in STA inhibiting M2 macrophage polarization, providing a new basis for understanding the molecular mechanism of STA, which is different from the signal pathways focused on in other studies. |
| Effect of activating the JAK2/STAT3 pathway on STA's inhibitory effect | a. After treatment with the JAK2/STAT3 activator Broussonin E, the inhibitory effect of STA on macrophage M2 polarization was reversed. b. In cell migration and invasion experiments, after activating the JAK2/STAT3 signaling pathway, the ability of STA to inhibit the migration, invasion, and angiogenesis of CRC cells and HUVECs induced by M2 macrophages was weakened. | **Similarities**: This research on the change of STA function after activating or inhibiting signal pathways is consistent with the current research idea of the relationship between signal pathways and drug effects and is similar to the exploration of signal pathway regulation of drug function in other studies. **Differences**: This study specifically studied the effect of activating the JAK2/STAT3 pathway on STA's inhibition of M2 macrophage-related functions, and the experimental design and observation indicators are unique. |
| Effect of STA combined with anti - PD - 1 treatment | - The combination treatment significantly inhibited tumor growth in mice, almost completely inhibited liver metastasis, reduced the number of liver metastases, decreased the liver/body weight ratio, and improved the survival rate of mice. - Flow cytometry analysis showed that the combination treatment inhibited the proportion of M2 macrophages in metastases, increased the proportion of CD8 ^+^ T cells, especially the proportion of CD8 ^+^ IFN - γ ^+^ T cells, enhanced the immunotherapeutic effect, and changed the tumor microenvironment from immunosuppression to immune activation. | **Similarities**: Consistent with the current research trend of combined tumor immunotherapy, such as the exploration of the synergistic effect of different drugs combined with immune checkpoint inhibitors in other studies, all focusing on the advantages of combination therapy in tumor treatment. **Differences**: This study provided experimental evidence for the application of STA in combined immunotherapy and specifically studied the effect of the combination of STA and anti - PD - 1 in the colorectal cancer liver metastasis model. Other studies may combine different drugs and target different tumor models. |
