# Supplementary figures and images for: Stachydrine targeting tumor-associated macrophages inhibit colorectal cancer liver metastasis by regulating the JAK2/STAT3 pathway
Source: Front Pharmacol. 2025 Feb 5;16:1514158. doi: 10.3389/fphar.2025.1514158 (PMC11835834; doi:10.3389/fphar.2025.1514158)

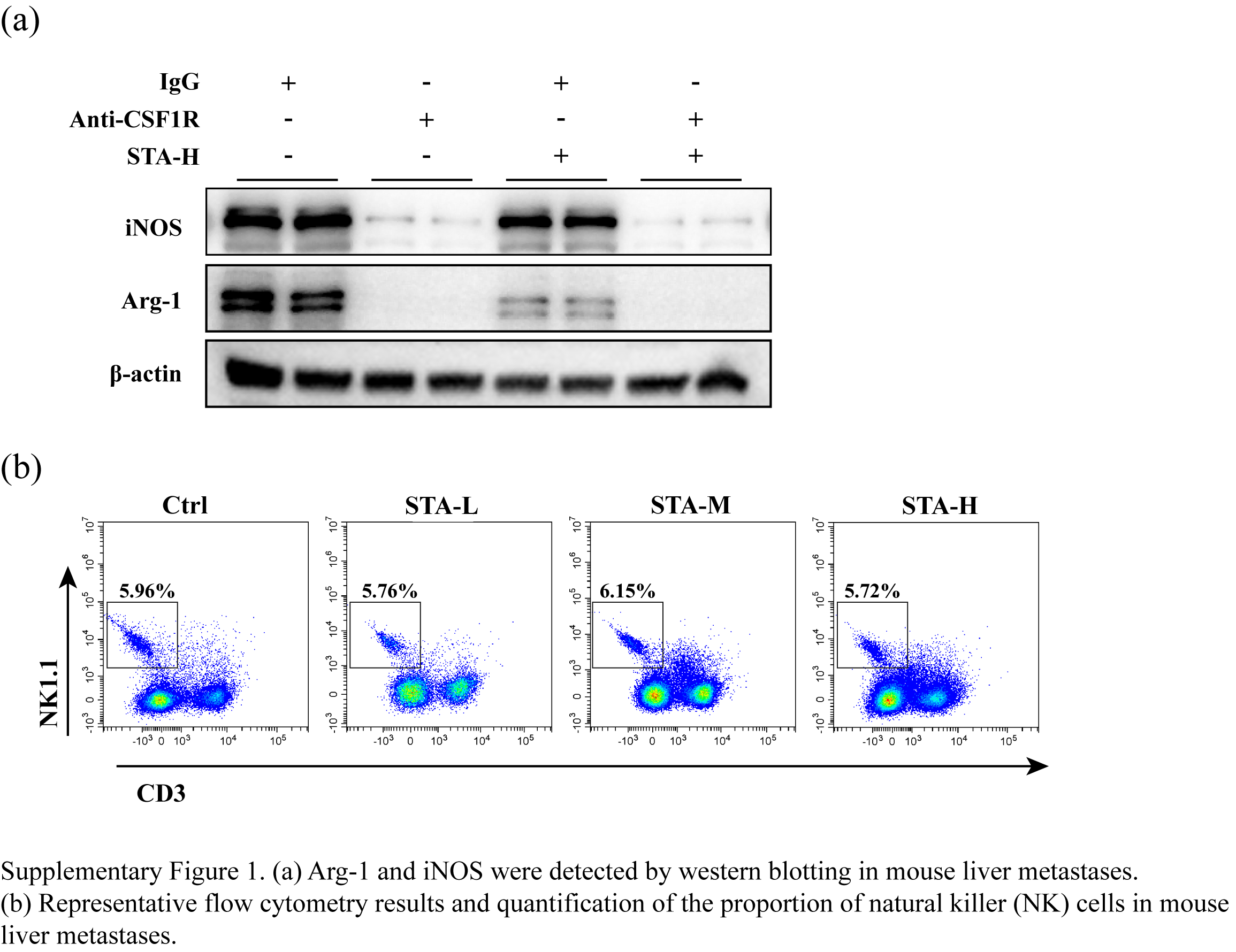

Supplement: Supplementary file 2 [file Image1.tif]
