# Supplementary material for: Stachydrine targeting tumor-associated macrophages inhibit colorectal cancer liver metastasis by regulating the JAK2/STAT3 pathway
Source: Front Pharmacol. 2025 Feb 5;16:1514158. doi: 10.3389/fphar.2025.1514158 (PMC11835834; doi:10.3389/fphar.2025.1514158)
Supplement: Supplementary file 3 [file Table2.docx]

| Supplementary Table 2. list of abbreviations | |
| --- | --- |
| Agr1 | Arginase 1 |
| ALT | Alanine transaminase |
| AST | Aspartate aminotransferase |
| BMDMs | Bone marrow-derived macrophages |
| CM | Conditioned medium |
| CRC | Colorectal cancer |
| CRLM | Colorectal cancer liver metastases |
| HUVECs | Human umbilical vein endothelial cells |
| ICB | Immune checkpoint blockade |
| M-CSF | Macrophage colony-stimulating factor |
| PD-L1 | Programmed Death-Ligand 1 |
| qRT-PCR | quantitative reverse transcription polymerase chain reaction |
| STA | Stachydrine |
| TAMs | Tumor-associated macrophages |
| TME | Tumor microenvironment |
