# Supplementary material for: Stachydrine targeting tumor-associated macrophages inhibit colorectal cancer liver metastasis by regulating the JAK2/STAT3 pathway
Source: Front Pharmacol. 2025 Feb 5;16:1514158. doi: 10.3389/fphar.2025.1514158 (PMC11835834; doi:10.3389/fphar.2025.1514158)
Supplement: Supplementary file 4 [file Table3.docx]

| Supplementary Table 3: Experimental reagent | |
| --- | --- |
| Stachydrine (STA) | S-078, purity＞98%, herbpurify |
| Anti-CSF1R Antibody | BioXCell, BE0213 |
| Anti-PD-1 Antibody | BioXCell, BE0146 |
| DMEM Medium | Gibco |
| Fetal Bovine Serum (FBS) | ExCell |
| Penicillin-Streptomycin | 15140122, Thermo |
| D-Luciferin Potassium Salt | 115144-35-9, Meilunbio |
| Collagenase V | C9263, Roche |
| Hyaluronidase | H1136, Roche |
| DNase I | 10104159001, Roche |
| Red Blood Cell Lysis Buffer | 420301, Biolegend |
| Leukocyte Activation Cocktail | BD, 550583 |
| Mouse Fc Receptor Blocker | BD, 553141 |
| Fixation/Permeabilization Kit | BD, 554714 |
| Fixable Viability Stain - BV510 | BD, 564406 |
| CD45 - APC/Cyanine7 | Biolegend, 103115 |
| CD45 - APC | BD, 561018 |
| F4/80 - PE | BD, 565410 |
| CD11b - Percp/Cyanine5.5 | Biolegend, 101227 |
| CD11c - BV421 | Biolegend, 117329 |
| CD206 - APC | Invitrogen, 2073756 |
| CD3e - PE | eBioscience, 12-0031-82 |
| CD4 - APC/Cyanine7 | BD, 552051 |
| CD8a - FITC | eBioscience, 11-0081-82 |
| IFN-γ - Alexa Fluor 647 | BD, 557735 |
| DAPI Staining | R37606, Thermo |
| Macrophage Colony-Stimulating Factor (M-CSF) | 315-02, Peprotech |
| Interleukin-4 (IL-4) | 214-14, Peprotech |
| RNAiso Plus | TaKaRa, 9109 |
| HiScript III 1st Strand cDNA Synthesis Kit | R312-01, Vazyme |
| RT-PCR Kit for Real-Time Fluorescence Quantitative PCR System | Q311-02, Vazyme |
| RIPA Lysis Buffer | P0013K, Beyotime |
| Polyvinylidene Difluoride (PVDF) Membrane | R1SB98889, Millipore |
| Blocking Solution | G2052, Servicebio |
| iNOS Antibody | #131205, Cell Signaling Technology, Inc. |
| Arg-1 Antibody | #93668, Cell Signaling Technology, Inc. |
| Phospho-Stat3 (Tyr705) Antibody | #9145, Cell Signaling Technology, Inc. |
| STAT3 Antibody | #9139, Cell Signaling Technology, Inc. |
| Phospho-Jak2 (Tyr1007/1008) Antibody | #3771, Cell Signaling Technology, Inc. |
| JAK2 Antibody | #3230, Cell Signaling Technology, Inc. |
| β-actin Antibody | #4967, Cell Signaling Technology, Inc. |
| Anti-Rabbit IgG, HRP-Linked Antibody | #7074, Cell Signaling Technology, Inc. |
| Anti-Mouse IgG, HRP-Linked Antibody | #7076, Cell Signaling Technology, Inc. |
| ECL Western Blotting Detection Reagent | 32209, Thermo |
| Matrigel | #E1270, Sigma, Missouri, America |
